# Clinical importance of laboratory biomarkers in liver fibrosis

**DOI:** 10.11613/BM.2022.030501

**Published:** 2022-10-01

**Authors:** Goda Aleknavičiūtė-Valienė, Valdas Banys

**Affiliations:** 1 Center of Laboratory Medicine,Vilnius University Hospital Santaros Klinikos, Vilnius, Lithuania; 2 Department of Physiology, Biochemistry, Microbiology and Laboratory Medicine, Faculty of Medicine, Vilnius University, Vilnius, Lithuania

**Keywords:** biomarkers, hepatic fibrosis, hyaluronic acid, collagen, laminin, cholylglycine

## Abstract

Hepatic cirrhosis is a major health problem across the world, causing high morbidity and mortality. This disease has many etiologies, yet the result of chronic hepatic injury is hepatic fibrosis causing cirrhosis and hepatocellular carcinoma, as the liver’s architecture is progressively destroyed. While liver biopsy is currently the gold standard for fibrosis staging, it has significant disadvantages, leading to a growing interest in non-invasive markers. Direct biomarkers – hyaluronic acid, laminin, collagen type III N-peptide, type IV collagen and cholylglycine – are new and rarely applied in routine clinical practice. This is the case primarily because there is no general consensus regarding the clinical application and effectiveness of the individual biomarkers. The usage of these markers in routine clinical practice could be advantageous for patients with liver fibrosis, requiring a simple blood test instead of a biopsy. The former option would be especially attractive for patients who are contraindicated for the latter. This review summarizes recent findings on direct biomarkers of liver fibrosis and highlights their possible applications and potential benefit for liver fibrosis diagnostics and/or staging.

## Introduction

Hepatic cirrhosis is a major health problem across the world, causing high morbidity and mortality. There are approximately 2 million deaths *per* year worldwide from cirrhosis (*1*). Cirrhosis is the last stage of liver fibrosis, in which the liver’s architecture is destroyed. This disease has many etiologies, such as alcoholic disease, non-alcoholic fatty liver disease (NAFLD), chronic viral hepatitis and cholestatic liver disease. Cirrhosis can be prevented by detection of liver fibrosis at an early stage and before the beginning of clinical symptoms. The METAVIR scoring system, which assigns a score ranging from no fibrosis (F0) to cirrhosis (F4), is the most commonly used scoring tool in Europe. While the liver biopsy is the gold standard for fibrosis staging, it has significant disadvantages, such as being a highly invasive procedure; high cost; potential complications such as pain, infection, peritoneal bleeding. Another disadvantage is that the histological distribution of fibrosis within liver parenchyma is heterogeneous and the diagnosis of fibrosis is based on a 15 mm biopsy specimen that reflects only 1/50,000 part of the liver, thus biopsies from different areas can show different stages of fibrosis; histological evaluation strictly depends on the experience of the pathologist (*2*). Diagnosis and determination of the stage of liver fibrosis is crucial both for cirrhosis risk evaluation as well as for its treatment. Non-invasive alternatives, such as FibroScan transient elastography, are widely used to assess fibrosis and steatosis. While FibroScan is a quick and safe method, there are some related drawbacks. There is limited access to FibroScan devices, especially in lower-income countries and such an assessment approach has technical limitations. It is of limited use with patients who have ascites, large amounts of chest wall fat, or individuals who are morbidly obese (*3*). As a consequence, there has been growing interest in non-invasive markers which may offer acceptable and cost-effective alternatives for both the patient and the specialist. This review summarizes recent findings on direct biomarkers of liver fibrosis and highlights their possible applications and potential benefit for liver fibrosis diagnostics and/or staging.

## Role of direct biomarkers in hepatic injury

The extracellular matrix (ECM) is essential for cell proliferation, migration and differentiation (*4*). The ECM’s secretion starts at the embryonic stage and is crucial for intrahepatic specification and maturation during liver development and regeneration (*5*). The development of hepatic fibrosis starts when the balance of deposition and removal within the extracellular matrix is disturbed (*5*). The ECM is mainly produced by hepatic stellate cells, which transdifferentiate into myofibroblast-like cells when injury and inflammation occur in the liver (*4*). Proteins of ECM– direct biomarkers – are soluble or secreted proteins, whose concentration is elevated in serum with hepatic fibrosis progression and decreases when the treatment is started (*6*, *7*).

There is growing interest in the clinical application of five biomarkers in hepatic fibrosis and cirrhosis: hyaluronic acid (HA), laminin (LN), collagen type III N-peptide (PIIIP N-P), type IV collagen (CIV) and cholylglycine (CG). A summary of their relevance to various etiologies is outlined in Table 1. Although, there are more biomarkers of interest, such as matrix metalloproteinase (MMP), tissue inhibitor of metalloproteinase I (TIMP-I), transforming growth factor β (TGF-β) and others. However, the markers considered within this review can all be tested using one straightforward system of analysis, which could reduce costs for the laboratory.

**Table 1:** The summary of biomarkers relevance in various etiologies

**Biomarker**	**Etiology**	**Relevance**	**Reference**
HA	HBV, HCV, AC, NAFLD	Liver fibrosis stage evaluation by increased HA concentration	(*12*, *13*, *20*, *21*, *27*, *32*, *33*)
HA	HBV, HCV, NAFLD	Healthy patients screening; differentiation from patients with liver fibrosis	(*14*, *16*, *24*, *28*, *30*)
HA	HBV	Monitoring of antiviral treatment	(*17*, *18*)
LN	HCV, HBV	Liver fibrosis stage evaluation by increased LN concentration	(*12*, *40*-*43*)
LN	HBV	Monitoring of antiviral treatment	(*46*)
PIIIP N-P	HBV, AC	Healthy patients screening; differentiation from patients with liver fibrosis	(*49*-*51*)
PIIIP N-P		Screening for MTX induced hepatic fibrosis	(*53*, *54*)
CIV	HCV, HBV	Liver fibrosis stage evaluation by increased CIV concentration	(*41*, *59*)
CIV	HBV, NASH	Healthy patients screening; differentiation from patients with liver fibrosis	(*50*)
CG	NA	Healthy patients screening; differentiation from patients with liver fibrosis	(*62*)
HA - hyaluronic acid. LN - laminin. PIIIP N-P - collagen type III N-peptide. CIV - type IV Collagen. CG - cholylglycine. HBV - hepatitis B virus, HCV - hepatitis C virus. AC - alcoholic cirrhosis. NAFLD - non-alcoholic fatty liver disease. MTX - methotrexate. NASH - non-alcoholic steatohepatitis. NA – not available.			

### Hyaluronic acid (HA)

Hyaluronic acid is among the most studied direct biomarkers. K. Meyer and J. Palmer discovered HA in 1934, in the vitreous of cows’ eyes (*8*). Unsurprisingly, the first medical application of HA was for eye surgery. Later its use was extended to various medical fields such as dermatology, orthopedics, and cardiology. Since 1985, HA has been employed for the differentiation of stages of liver disease.

The HA molecule is a glycosaminoglycan of high molecular weight, composed of a repetitive sequence of hexuronic and amino sugars with acetyl groups. In every molecule the number of disaccharides is 2000-25,000, thus molecular weight varies from 105 to 107 Da. It is one of the most hydrophilic molecules in the human body, binding water and controlling the hydration of tissues. Hyaluronic acid can be found freely in the lymphatic system, in blood circulation, in the ECM and bound to receptors on cell surfaces (*9*). It is produced by activated hepatic stellate cells (HSC) and is the leading component of the ECM. Hyaluronic acid synthases synthesize the HA molecules by adding activated substrate forms to the growing chain, *i.e.,* UDP-glucuronic acid and UDP-acetylglucosamine (*10*). These molecules go through the plasma membrane and are secreted into the extracellular space (*9*). The uptake and degradation of HA occur in hepatic sinusoidal endothelial cells. In healthy liver serum, concentrations of HA are low as circulating HA is speedily eliminated from the blood mainly by the liver, but also by the spleen and kidneys. In blood the half-life of HA is 2-5 minutes but in a damaged liver an increasing concentration in serum is observable. This is due to the increased production of HA and the decreased hepatic elimination of HA which is indicative of the fibrosis stage (*7*).

In 1994, Gallorini *et al.,* defined the upper limit of the normal range as 98 µg/l (*11*). Since then, many different studies have been conducted on the use of HA biomarkers in various etiologies of liver fibrosis. The newest studies are described in Table 2. Several studies have been conducted on using serum HA for differentiating fibrosis in patients with chronic hepatitis B virus (HBV). Li *et al.* concluded that the HA concentration in serum significantly increased depending on the stage of liver fibrosis, thereby establishing a positive correlation with the stages of fibrosis (*12*). Montazeri *et al.* showed that the HA in serum is an effective biomarker for the evaluation of stages of fibrosis in patients who have HBV infection (*13*). A 2010 study confirmed that the serum concentration of HA significantly increased in HBV infected patients, compared with healthy individuals (*14*). Geramizadeh *et al.* reported that HA concentration is highest in severe fibrosis patients with HBV (*15*). Authors concluded that HA biomarker can exclude severe fibrosis and cirrhosis in HBV patients. Khan *et al.* showed that the mean of the serum HA in patients diagnosed with HBV was almost 10-times higher compared with the control group of healthy individuals and the difference was statistically significant (*16*). In patients with stage 4 fibrosis there was a significantly higher HA mean. This study further supports the proposition that serum HA concentration rises with the stage of liver fibrosis in patients with chronic liver disease. Furthermore, in a study of 60 HBV infected patients, who received 12 months of entecavir therapy, HA concentration was halved (*17*). A 2006 study showed that in children with hepatic fibrosis caused by HBV infection, who received interferon alpha treatment, HA concentration significantly decreased after 12 months of treatment (*18*). These studies support the proposition that monitoring the concentration of HA is appropriate for assessing the response to HBV infection treatment.

**Table 2:** Summary of performance characteristics of biomarkers in cirrhotic patients (according to Jothimani *et al*. (*69*))

**Biomarker**	**NPV, %**	**PPV, %**	**Sensitivity, %**	**Specificity, %**	**95% CI**	**Cut-off**
CG	98	98	97	96	NA	NA
HA	93	98	96	97	NA	NA
CIV	87	98	92	97	NA	NA
PIIIP N-P	86	98	92	96	NA	NA
LN	78	95	87	96	NA	NA
95% CI for any of the characteristics (NPV; PPV; specificity; sensitivity). Since 95% CI values are not available, no reliable conclusions could be obtained and the given biomarkers results are only indications. NPV - negative predictive value. PPV - positive predictive value. 95% CI - 95% confidence interval. NA - not available. CG - cholylglycine. HA - hyaluronic acid. CIV - type IV Collagen. PIIIP N-P - collagen type III N-peptide. LN - laminin.						

In 1996, Guéchot *et al.,* reported that HA is an important biomarker for the indication of cirrhosis in patients with HCV infection (*19*). Since then, more studies have been undertaken on variation in HA concentrations in patients with HCV. Abd-Elghany *et al.* confirmed that the concentration of HA rises along with the progression of stages of liver fibrosis in patients with the HCV infection (*20*). Another study confirmed that the concentration of HA increases significantly with the advancing stages of fibrosis in patients with HCV and with a change in the histologic activity index (*21*). These studies support the proposition that the HA biomarker is suitable for the differentiation of HCV caused hepatic fibrosis stages. Furthermore, in patients with HCV caused cirrhosis, concentrations of HA correlate with clinical severity, stiffness of liver and with the activity of the disease (*22*, *23*). McHutchinson *et al.* showed that the HA biomarker can be used to exclude cirrhosis or advanced fibrosis (*24*). Moreover, in comparisons with the aspartate aminotransferase (AST)/platelet ratio (APRI) and the widely used AAR AST/alanine aminotransferase (ALT) ratio (AAR) in patients with HCV, HA was the most effective marker for the diagnosis of fibrosis. This illustrates the potential of biomarkers to offer better diagnostic performance in clinical laboratories (*25*).

Non-alcoholic fatty liver disease is divided into two types: non-alcoholic fatty liver (NAFL), and non-alcoholic steatohepatitis (NASH). Both of these conditions can progress into fibrosis and/or cirrhosis. According to Mizuno *et al.,* HA is not a very efficient biomarker to distinguish between NASH and NAFL in an onset stage (*26*). Nevertheless, Dvorak *et al.* showed that the concentration of HA is higher in patients with advanced fibrosis compared to mild fibrosis (*27*). The study’s authors concluded that HA biomarker can differentiate patients with NASH and/or advanced fibrosis from those with simple steatosis. Lebensztejn *et al.* confirmed that HA is elevated in children with NAFLD and can serve to differentiate between patients with and without fibrosis (*28*). ElGhandour *et al.* proposed that HA could be employed as a direct biomarker for NASH. The authors found that this biomarker offered impressive performance in aiding the assessment of a fatty liver (*29*). Similarly, for NASH, the diagnostic performance of HA was excellent. The authors concluded that HA could be used as an accurate and reliable marker for the diagnosis of NASH. Baranova *et al.* describe a study of NAFLD related fibrosis, showing that the negative predictive value (NPV) is much higher than the positive predictive value (PPV). Therefore, they maintained, HA can be used to rule out advanced fibrosis and cirrhosis (*30*).

Variation in HA concentration has been studied in other liver fibrosis etiologies. One study found that the concentration of HA in serum is higher in alcoholic cirrhosis (AC), non-alcoholic cirrhosis (NAC) and toxic hepatitis (HT) compared with a control group (*31*). A statistically significant difference was determined between AC and NAC. The study’s authors concluded that the best diagnostic performance of HA was in AC. Other studies which measured the concentration of HA in serum in patients with AC, confirmed that concentration increased with the severity of liver fibrosis. Thus, these studies concluded that this biomarker could be used as indicator for cirrhosis (*32*, *33*).

There is a substantial body of research on the diagnostic performance of HA for different etiologies. However, Plevris *et al.* showed that analysis of HA concentration in serum performed independently of etiology and can be used for patients with varying etiologies and severities of liver disease (*34*). Overall, there is a high variability not just between recommended cut-off values among studies, but also between other statistical parameters such as NPV and PPV. Consequently, to choose a cut-off value in practice is challenging, as there is no general agreement on which value should be used, and whether the values should differ for different etiologies. The variation in recommended cut-off values can be explained by the fact that while choosing the best threshold to maximize sensitivity or specificity, the accuracy of one is sacrificed for the other (*35*). Moreover, to be able to evaluate biomarker specificity and sensitivity performance, it is crucial to have 95% confidence intervals (95% CI). Unfortunately, none of the conducted studies provide these values. While many studies have been conducted on the clinical performance of HA for different etiologies, there is an absence of strong evidence to prove that HA is a good diagnostic biomarker.

### Laminin (LN)

Laminin was first described by Timpl and Martin (1979) in murine fibrosarcoma (*36*). It is a non-collagenous glycoprotein which is synthesized by HSC and deposited in the liver’s basement membrane. It is a large complex comprised of 3 chains (α1, β1, γ1), of about 850 kDa in total. Receptors of this molecule are on the surface of many cells: platelets, muscle cells, neutrophils, and hepatocytes. The molecule’s main functions include: cellular adhesion; binding to collagens and glycosaminoglycans as matrix composition; and the maintenance of cytoskeleton and fibrogenesis mechanisms (*37*). In a healthy liver, LN is found around the vessels and biliary ducts, while in a liver with cirrhosis LN deposition appears in the space of Disse (*36*). Consequently, LN elevation in serum can be an indicator of chronic liver injury since architectural changes in liver parenchyma can lead to liver fibrosis.

Sebastiani *et al.* noted that several HCV studies have described normal aminotransferase activities in 25-30% of chronic HCV patients. Thus, indirect serum markers of fibrosis in chronic HCV patients reflect changes in hepatic function and not in ECM metabolism (*38*). Consequently, a more specific biomarker with greater diagnostic performance is required. In 1991, Kropf *et al.* showed that LN can be used as a screening test for hepatic fibrotic disease (*39*). Further studies have shown that in patients with HBV or HCV infection, LN can be used to evaluate fibrosis. As damage to liver endothelial cell function leads to an increase in LN concentration in serum, there exists a correlation with LN concentrations and the stage of hepatic fibrosis (*12*, *40*-*42*). Hafez *et al.* showed that LN can be used for the identification of fibrosis in patients with HBV, an important finding for patients when biopsy is contraindicated (*43*). El-Saeid *et al.* illustrated that in patients with chronic hepatitis B and C there is a positive correlation between LN and the stage of liver fibrosis (*44*).

Yongdi *et al.,* in a 2019 meta-analysis study of LN in HBV infected patients, showed that elevated concentration of LN in serum indicates an increased risk of liver fibrosis (*45*). Patients with an elevated concentration of LN in serum could be closely monitored and receive early treatment to prevent the development of liver fibrosis. After six months of treatment with interferon, adefovir or lamivudine, the LN concentration in serum was observed to decrease (*46*). The treatment stimulates the regeneration of endothelial cells in the liver allowing new cells to metabolize laminin more effectively. This research illustrates the potential of employing laminin as a biomarker to effectively monitor treatment progression.

The majority of studies primarily focus on the diagnostic performance of LN in HBV infected patients. There is a high level of variation across these studies as regards cut-off values and other statistical parameters such as NPV and PPV. As is the case with studies of HA, none of the LN studies provide 95% confidence intervals.

### Collagen type III N-peptide (PIIIP N-P)

In 1979, Rojkind *et al.* found that in cirrhotic liver the collagen content is elevated by 4 to 7 times that found in a healthy liver, with two main types of collagen present (types I and III) (*47*). Collagen type III N-peptide is one of the largest ECM components in the liver. *Via* the type III collagen synthesis, the N-terminal propeptide of procollagen type III is removed from procollagen type III, resulting in the release of this molecule into the blood (*48*). This molecule is a component of connective tissue and its concentration in serum rises in hepatic fibrogenesis.

In early 1988, Zanten *et al.* evaluated the diagnostic application of PIIIP N-P in alcoholic liver disease and found that this marker was significantly elevated in patients with AC (*49*). Hasan *et al.* evaluated the diagnostic accuracy of the PIIIP N-P marker in patients with chronic HBV (*50*). The authors evaluated biomarker specificity and sensitivity and concluded that PIIIP N-P could be used to differentiate patients with chronic HBV from healthy individuals. Tang *et al.* conducted a clinical trial on serum biomarkers of liver fibrosis in infants with cholestasis (*51*). They confirmed that PIIIP N-P values were significantly higher (P < 0.010) in infants with cholestasis than in healthy individuals. This study indicated the effectiveness of utilizing PIIIP N-P in differentiating healthy individuals from those with hepatic fibrosis. However, Kader *et al*. study (with a small sample size) revealed that there was no significant difference in PIIIP N-P concentration between mild, moderate and severe fibrosis in patients with chronic hepatitis B and C (*52*). As a consequence, the authors concluded that this biomarker cannot replace liver biopsy and cannot be used in differentiating the stage of fibrosis.

In addition, long-lasting methotrexate (MTX) therapy can cause fatty liver, hepatic fibrosis and cirrhosis development. Usually, fatty liver and hepatic fibrosis are asymptomatic until cirrhosis is present and routine laboratory liver function tests do not indicate abnormal or significant elevation. Lotfy *et al.* stated that the serum PIIIP N-P biomarker can detect liver fibrosis and could be used in screening patients on long term MTX (*53*). Notably, the British Association of Dermatologists recommend the use of PIIIP N-P in adults before starting the MTX treatment for moderate-to-severe psoriasis and at 3-month intervals throughout the treatment. Therefore, PIIIP N-P can be used as screening non–invasive marker for MTX induced hepatic fibrosis (*54*).

The expression of PIIIP N-P is restricted to soft tissues and correlates with the number of myofibroblasts in fibrotic tissue. Crucially, this marker is not liver specific; its presence increases with the progress of other diseases, such as lung fibrosis, acromegaly, rheumatoid diseases and chronic pancreatitis (*55*, *56*). Recognizing these limitations is important to understanding this biomarker’s diagnostic utility.

### Type IV collagen (CIV)

Another molecule, which has aroused interest as regards the evaluation of liver injury is type IV collagen. This type of collagen is a basement membrane component and reflects its regeneration. The collagen family is a group of proteins consisting of 28 different types, with a highly stable triple helix structure including three constituent chains that have a repetitive core amino acid sequence (glycine-proline-hydroxyproline) (*57*). Type IV collagen is present in healthy livers, supporting specialized polarized cells. It forms a low-density basement membrane-like matrix along the sinusoid, bile ducts and vessels of the portal tract. In comparison to types I and III collagens, which are partly processed proteolytically, CIV remains intact in the matrix and is composed of six alpha chains α1-6, which form heterotrimers α1α1α2, α3α4α5, and α5α5α6 (*58*).

There is limited research on this biomarker’s performance in hepatic fibrosis. In patients with HCV or HBV infection, the concentration of CIV increased significantly with the stage of fibrosis, compared with the traditional markers ALT, alkaline phosphatase (ALP) and bilirubin, which did not differ significantly (*41*, *59*). A 2021 study confirmed that CIV has the potential to be used in the clinical laboratory for the detection of fibrosis in patients with HBV (*50*). Mizuno *et al.* showed that the expression of the CIV marker is significantly increased in patients with NASH and that it can be a useful marker in the evaluation of NASH severity (*26*).

### Cholylglycine (CG)

Another molecule of interest for the evaluation of liver fibrosis is cholyglycine. Of all markers described here, the use of this molecule for the assessment of hepatic fibrosis is the least studied, with only limited research undertaken. Cholylglycine is synthesized in the liver cells as cholic acid which is conjugated with glycine and then transported to the gallbladder for storage *via* the bile duct (*60*). After every meal the gallbladder starts to contract and CG goes along with the bile into the small intestine, where it takes part in fat digestion and absorption. Then 95% of the bile is reabsorbed by the small intestinal mucosa into the blood and transported back to the liver. Enterohepatic circulation occurs 6-10 times *per* day (*61*). The CG concentration in peripheral blood is normally very low (< 2.65 mg/L), while in cases of liver damage, CG concentration in blood will increase. In a cirrhotic liver, the CG concentration in serum is about 10 to 100 times higher (*61*).

One of the few studies on CG’s performance as a biomarker, Tanggo *et al.,* revealed that CG concentration is elevated in patients with acute hepatitis and liver cirrhosis and could be used in screening individuals for cirrhosis (*62*). In a 2020 study, Liu *et al*., showed that a reusable optofluidic point-of-care testing platform could be successfully adapted for the measurement of CG concentration in serum, with the authors noting that it can offer quick, easy and early diagnosis (*63*).

### Simultaneous measurement of biomarkers

Li *et al.* studied the use of simultaneous measurements of HA and LN for identifying significant fibrosis (*12*). They discovered that this approach resulted in better PPV (100%) than when a single biomarker was measured. Seven *et al.* reported that simultaneous measurement of HA and TIMP-1 proves a reliable tool for the identification of advanced stage liver fibrosis induced by HBV, and can be used to complement information obtained from a liver biopsy (*64*). The enhanced liver fibrosis (ELF) test, measures three direct markers for the presence and stage evaluation of fibrosis, and presents a score calculated according to an algorithm. The markers are HA, PIIIP N-P, and TIMP-1, and while this test retains the same title, there are three different formulas of this algorithm (Guha and two Siemens), which produce highly correlated results (*65*). The ELF test enables the detection of fibrosis and rules out significant fibrosis for a wide range of etiologies including: NAFLD, HCV, HBV, MTX induced liver fibrosis, and AC (*66*, *67*). While the ELF test is prognostic and disease-monitoring, it is recommended by the National Institute for Health and Care Excellence for the management of non-alcoholic fatty liver disease (*68*). However, a recent study shows that while the ELF test is highly sensitive, it offers limited specificity to exclude advanced and significant fibrosis at low cut-off values in patients with NAFLD (*65*). The authors therefore, concluded that it is important to adopt suitable test thresholds to achieve the desired performance.

### Comparison of five biomarkers

Five hepatic markers of fibrosis - HA, LN, PIINP, CIV and CG - are new and rarely utilised in routine clinical practice. Snibe Diagnostic is one of the few manufacturers who offer all five tests of biomarkers on one analyser, with the chemiluminescence immunoassay (CLIA) system – Maglumi.

In 2018, Jothimani *et al.* performed a comparison study of these five biomarkers using the Snibe Maglumi analyzer (*69*). The results indicated that all the markers’ concentration in serum were statistically significantly higher in the cirrhosis group than in the control one (P < 0.001) (Table 2). However, which biomarker showed the best diagnostic values is impossible to state since this research is missing the 95% Cl – a crucial parameter that could prove that. Moreover, it is important to note that the lack of 95% CI is critical since no proper validation of the results cannot be conducted without it and no plausible and reliable conclusions could be obtained. A study by Stefano *et al.,* published in 2021, evaluated all five biomarkers using the Snibe analyser in patients with NAFLD (*70*). The study revealed that a CIV concentration above 30 ng/mL indicated a greater possibility of significant and advanced fibrosis. It was the only marker with a statistically significant result, while for other markers – HA, PIIIP N-P, HA, LN – the chosen cut-off values did not detect the presence of significant and advanced fibrosis. Therefore, it appears that CIV can identify the presence of significant and advanced fibrosis in patients with NAFLD. Nevertheless, in order to employ this approach as part of routine clinical practice further research is required.

## Conclusion

Liver fibrosis is an increasingly common global health problem and its diagnostics remain highly invasive for patients as there are no biomarkers with good diagnostic performance for early screening or even stage evaluation. This review surveys the analyses of five emerging direct biomarkers of liver fibrosis, which could potentially replace liver biopsy. The main advantages of these biomarkers are that: drawing blood is less invasive than a liver biopsy, laboratory tests are easy applicable, tests for biomarkers have good reproducibility and could be performed in most of the laboratories, and allow for the evaluation of the pathophysiologic progress and processes. However, there are several disadvantages: most of these markers do not differentiate between the intermediate stages, none of the biomarkers are liver-specific, results can be affected by comorbidities, and often they have limited analytical accuracy (*10*).

There is still no general agreement as to which single biomarker or simultaneous measurement of biomarkers is most suitable for screening or staging of liver fibrosis. To begin using these biomarkers in accurate diagnostics, treatment and prevention of fibrosis in the patients with liver disease, would require clarity on which biomarker is the most effective and consensus on cut-off values. At the moment, HA shows promising results in fibrosis stage evaluation and screening, especially in patients with HBV, HCV and NAFLD. The LN biomarker could be effectively used in staging of liver fibrosis for HBV and HCV patients. Both biomarkers – HA and LN – have potential for monitoring of antiviral treatment in patients with HBV. The PIIIP N-P biomarker could be used for screening for MTX induced liver fibrosis as well as HBV patients with liver fibrosis. The CIV biomarker shows promising results for fibrosis staging in patients with HCV or HBV infection. As little research has been conducted on the CG biomarker, no reliable conclusions can be made. Although, there is evidence of the significant clinical utility of these biomarkers, all of the studies lack crucial statistical information such as 95% Cl for specificity, sensitivity and other parameters. As a consequence, there is no strong, reliable data about the diagnostic accuracy of these biomarkers. As present studies results are only indicating, but not providing these biomarkers value in consideration, further research and validation are required before any systematic introduction of their use into clinical practice is considered. Specifically, more effective collaboration between hepatologists and laboratory medicine specialists is necessary to transform promising diagnostic results offered by biomarkers into effective routine clinical tests.

## Data Availability

No data was generated during this study, thus a data sharing statement is not applicable.
